# Labor Induction in Obese Pregnancies at Term: Risk Factors and Pregnancy Outcomes

**DOI:** 10.1155/jp/8982438

**Published:** 2026-03-31

**Authors:** Huwaida Nabilah, Hermanto Tri Joewono, Muhammad Ilham Aldika Akbar

**Affiliations:** ^1^ Department Obstetrics & Gynecology, Faculty of Medicine Universitas Airlangga, Surabaya, Indonesia; ^2^ Department Obstetrics & Gynecology, Dr. Soetomo General Academic Hospital, Surabaya, Indonesia, rsudrsoetomo.jatimprov.go.id; ^3^ Department Obstetrics & Gynecology, Universitas Airlangga Academic Hospital, Surabaya, Indonesia

**Keywords:** Bishop score, labor induced, obesity maternal, pregnancy outcome

## Abstract

**Objectives:**

The objectives of this study are to identify risk factors associated with failed labor induction in obese pregnancies at term and evaluate related maternal and neonatal outcomes.

**Methods:**

This cross‐sectional study included obese pregnant women (BMI ≥ 30 kg/m^2^) who underwent labor induction at 37–41 weeks′ gestation in two academic hospitals in Surabaya, Indonesia, during 2023. Participants were classified into successful and failed induction groups. Maternal characteristics, induction‐related factors, and pregnancy outcomes were compared. Multivariable logistic regression was used to identify independent risk factors for induction failure.

**Results:**

A total of 159 women were included, comprising 53 failed and 106 successful inductions. Failed induction was significantly associated with primiparity (adjusted odds ratio [aOR], 3.10; 95% CI, 1.46–6.58), Bishop score < 3 (aOR, 2.02; 95% CI, 1.14–3.55), and higher maternal weight gain during pregnancy (aOR per kg increase, 0.89; 95% CI, 0.84–0.95). Maternal weight gain demonstrated a moderate effect size (standardized mean difference, 0.62). Compared with successful induction, failed induction was associated with higher rates of severe preeclampsia (18.9% vs. 1.9%), cesarean delivery (100% vs. 5.7%), longer hospitalization, and lower 1‐min Apgar scores. No significant differences were observed in 5‐min Apgar scores, fetal growth restriction, congenital anomalies, or NICU admission.

**Conclusions:**

In obese pregnancies at term, primiparity, unfavorable cervical status, and excessive gestational weight gain are independently associated with failed labor induction. These findings highlight the importance of optimized patient selection, cervical assessment, and anticipatory counseling to improve maternal and neonatal outcomes.

## 1. Introduction

Obesity is an increasing global health concern affecting not only high‐income countries but also low‐ and middle‐income regions. According to the World Health Organization, obesity is defined as a body mass index (BMI) ≥ 30 kg/m [1], whereas overweight is defined as a BMI ≥ 25 kg/m [1]. In 2022, more than 890 million adults worldwide were classified as obese, and approximately 2.5 billion were overweight. In Southeast Asia, Indonesia has one of the highest burdens of overweight and obesity, affecting more than 30% of the adult population [2]. RISKESDAS (Indonesian national health research) estimated that in 2023 the number of obesity cases has reached 15.3% [1]. The prevalence of overweight and obesity is higher among women than men, with 44.4% of adult women and 11.3% of adult men affected in 2018 [3]. Royal College of Obstetricians and Gynecologists (RCOG) reported that the prevalence of obesity in pregnancy ranges from 16%–19% [4]. Globally, approximately 39 million pregnancies annually are complicated by maternal obesity, with certain countries reporting an estimated prevalence of overweight and obesity during pregnancy exceeding 60% (South Africa 64%, Mexico 65%, United States 55%–63%) [5].

Maternal obesity is associated with an increased risk of adverse pregnancy and labor outcomes. Complications among pregnant women with obesity include thromboembolism, preeclampsia, eclampsia, higher rates of labor induction, and induction failure. Fetal and neonatal risks include macrosomia, shoulder dystocia, and pregnancy loss [6]. Labor induction may be indicated in pregnancies complicated by maternal obesity to reduce the risks of postterm gestation and late‐term stillbirth, as well as to manage the higher burden of preexisting comorbidities and pregnancy‐related complications. Compared with expectant management, induction of labor at 39–40 weeks′ gestation has been associated with lower cesarean delivery rates and reduced maternal and neonatal morbidity. However, the likelihood of induction failure increases with greater severity of maternal obesity, and obese women are at heightened risk of cesarean delivery and prolonged labor courses secondary to failed induction [7]. There is some evidence to suggest that labor induction is less likely to be efficacious in pregnant women with an increasing BMI [8, 9]. The objective of this study was to compare risk factors and pregnancy outcomes between obese pregnancies with failed labor induction and those with successful induction.

## 2. Methods

This research is an analytic cross‐sectional study, using data collected from Universitas Airlangga Hospital (RS UNAIR) and Dr. Soetomo General Academic Hospital, Surabaya (Indonesia) from January to December 2023. Both institutions are teaching hospitals that are affiliated with the Faculty of Medicine Universitas Airlangga. RS UNAIR is classified as a secondary‐type teaching hospital, whereas Dr. Soetomo General Academic Hospital is a tertiary‐type teaching hospital that offers comprehensive subspecialist services. The ethical eligibility of this study has been approved by the Ethics Commission of Dr. Soetomo General Academic Hospital Surabaya (1027/KEPK/VII/2024) and Universitas Airlangga Hospital Surabaya (085/KEP/2024).

Eligible participants were obese pregnant women who underwent labor induction at 37–41 weeks′ gestation and met the predefined inclusion and exclusion criteria. Inclusion criteria comprised singleton pregnancies with cephalic presentation and no contraindication to vaginal delivery. Women with incomplete data or prelabor rupture of membranes were excluded. Sample size estimation using the Lemeshow formula indicated a minimum of 50 participants per group. Participants were enrolled consecutively and categorized into successful or failed labor induction groups.

The labor induction protocol was similar between both hospitals involved in this study. The labor induction protocol at our hospital comprises the administration of oxytocin at a dosage of 10 IU per 500 cc of Ringer lactate (RL) solution, administered twice, resulting in a total of 1000 cc of RL solution. This is referred to as a one/single series of labor induction. Oxytocin administration commences at a modest dosage of eight drops per minute, subsequently increasing by four drops every 15 min, until a maximum of 40 drops per minute is attained, continuing until the fluid is exhausted.

Successful labor induction was defined as progression to the active phase of labor or cervical dilation > 4 cm following a single induction series. Induction failure was defined as failure to reach the active phase or cervical dilation ≤ 4 cm after one induction series, or the occurrence of fetal distress before the onset of active labor. Maternal obesity was defined as a BMI ≥ 30 kg/m [1] in the third trimester and categorized as Class I (BMI 30–34.9 kg/m [1]), Class II (BMI 35–39.9 kg/m [1]), or Class III (BMI ≥40 kg/m [1]). The Bishop pelvic score (PS) was based on fetal station and cervical dilation, effacement, consistency, and position, and was dichotomized as < 3 or ≥ 310.

Risk factors assessed for failed labor induction included obesity class, maternal age, parity, Bishop score, estimated fetal weight, and gestational weight gain. Primary outcomes comprised induction success status (failed vs. successful), as well as maternal and neonatal outcomes. Maternal outcomes included pregnancy complications (pregestational diabetes, gestational diabetes, and hypertensive disorders of pregnancy), mode of delivery (vaginal vs. cesarean), and delivery‐related complications (postpartum hemorrhage, shoulder dystocia, and length of hospitalization). Neonatal outcomes included birth weight, 1‐ and 5‐min Apgar scores, incidence of small for gestational age, congenital anomalies, macrosomia, and NICU admission.

Data were analyzed using SPSS software. Categorical variables were compared using the chi‐square test, whereas ordinal variables were analyzed with the Mann–Whitney *U* test. Continuous variables were assessed for normality and compared using the independent‐samples *t* test or the Mann–Whitney *U* test, as appropriate. Multivariable associations were evaluated using logistic regression analysis.

## 3. Results

A total of 159 women met the inclusion and exclusion criteria, comprising 53 failed inductions and 106 successful inductions. Of these, 96 women (60.4%) received routine antenatal care at the study centers, whereas 63 (39.6%) were referred from other healthcare facilities. Most study participants were managed at Universitas Airlangga Hospital (87.4%) and resided in Surabaya (91.8%).

### 3.1. Risk Factor Associated With Failed Inductions

Risk factors assessed included maternal age, parity, fetal birthweight, Bishop score, degree of obesity, and gestational weight gain. Significant differences between failed and successful induction groups were observed for parity, Bishop score, and maternal weight gain (all *p* < 0.05). Women who experienced failed induction were more likely to be primiparous (58.5% vs. 26.4%; OR = 3.93; 95% CI, 1.96–7.88; *p* = 0.001) and to have an unfavorable Bishop score (PS < 3) at induction (73.6% vs. 45.3%; OR = 3.37; 95% CI, 1.64–6.91; *p* = 0.001).

Maternal weight gain was significantly higher in the failed induction group compared with the successful induction group (18.06 ± 5.97 vs. 14.30 ± 6.13 kg; *p* = 0.001), with a moderate effect size (standardized mean difference [Cohen^′^s d] = 0.62). No significant differences were observed between groups with respect to maternal age, fetal birthweight, induction agent, or obesity class (all *p* > 0.05) (Table 1). Variables that differed significantly on univariable analysis were entered into a multivariable logistic regression model. In adjusted analyses, primiparity emerged as the strongest independent predictor of failed labor induction in obese pregnancies, followed by an unfavorable Bishop score and greater gestational weight gain (Table 2).

**Table 1:** Risk factors associated with failed inductions.

Risk factors	Status of induction success		*p* value	OR value
	Failed *n*(%)	Success *n*(%)		
**Age**				
< 20 years	2 (3.8%)	4 (3.8%)		
20–34 years old	45 (84.9%)	80 (75.5%)	0.191	—
≥ 35 years old	6 (11.3%)	22 (20.8%)		
**Parity**				
Primipara	31 (58.5%)	28 (26.4%)	**0.001**	**OR: 3.93**
Multipara	22 (41.5%)	78 (73.6%)		**CI 95% (1.96–7.88)**
				**Contingency**
				**Coefficient (CC): 29.9%**
**Fetal Weight**				
≤ 2500 g	1 (1.9%)	7 (6.6%)		
2500–4000 g	50 (94.3%)	96 (90.6%)	0.238	—
≥ 4000 g	2 (3.8%)	3 (2.8%)		
**Bishop′s pelvic score**				
PS < 3	39 (73.6%)	48 (45.3%)		**OR: 3.37**
			**0.001**	**95% CI (1.64–6.91)**
PS ≥ 3	14 (26.4%)	58 (54.7%)		**CC: 26%**
**Degree of Obesity**				
Class I	35 (66.0%)	78 (73.6%)		
Class II	18 (34.0%)	18 (25.5%)	0.346	—
Class III	0 (0%)	1 (0.9%)		
**Pharmacology agent**				
Misoprostol	51 (96,2%)	99 (93,4%)	0.719	—
Oxytocin	2 (3,8%)	7 (6,6%)		
**M** **e** **a** **n** + **s** **t** **a** **n** **d** **a** **r** **d** **d** **e** **v** **i** **a** **t** **i** **o** **n**				
Maternal weight gain	18.06 + 5.97	14.30 + 6.13	**0.001**	—

*Note:* The bold number/values indicate statistically significant calculations (*p* < 0.05).

**Table 2:** Multivariable logistic regression analysis of factors associated with failed labor induction in obese pregnancies.

Variable	Adjusted OR	95% CI	*p* value
Primiparity	3.10	1.46–6.58	0.003
Bishop score < 3	2.02	1.14–3.55	0.015
Maternal weight gain (per kg increase)	0.89	0.84–0.95	0.001

*Note:* Adjusted for variables included in the multivariable model. Odds ratios represent the likelihood of failed labor induction.

### 3.2. Maternal Outcomes

Maternal outcomes differed between the successful and failed induction groups (Table 3). Failed induction was associated with a substantially higher incidence of severe preeclampsia (18.9% vs. 1.9%; OR, 2.85; 95% CI, 1.99–4.07; *p* = 0.001). No significant differences were observed in other maternal comorbidities, including preeclampsia, chronic hypertension, gestational hypertension, gestational diabetes, or pregestational diabetes. Mode of delivery differed markedly between groups, with all women in the failed induction group undergoing cesarean delivery (OR, 1654.34; 95% CI, 91.43–29,993; *p* = 0.001). In contrast, cesarean delivery occurred in 5.7% of women in the successful induction group, primarily due to fetal distress or labor arrest. The failed induction group experienced a longer stay in the hospital compared with the successful group (4 [3–7] days vs. 2 [1–5] days; *p* = 0.001).

**Table 3:** Maternal outcomes in pregnancies with obesity that failed and successfully induced labor.

Maternal outcome	Status of induction labor		*p* value	OR value
	Failed *N*(%) 53 (100%)	Success *N*(%) 106 (100%)		
**Pregnancy complications**				
Pregestational DM	2 (3.8%)	2 (1.9%)	0.601	—
Gestational DM	4 (7.5%)	15 (14.2%)	0.342	—
Chronic HT	3 (5.7%)	5 (4.7%)	1.000	—
Gestational HT	3 (5.7%)	8 (7.5%)	0.753	—
Preeclampsia (PE)	12 (22.6%)	16 (15.1%)	0.339	—
Severe PE	10 (18.9%)	2 (1.9%)	**0.001**	**OR 2.85** **CI 95% (1.99–4.07)** **CC: 29%**
**Delivery method**				
Vaginal delivery	0 (0%)	100 (94.3%)	**0.001**	**OR 1654.34** **95% CI (91.43–29993)** **CC: 67.7%**
Cesarean section	53 (100%)	6 (5.7%)		
**Labor complications**				
Shoulder dystocia	0 (0%)	2 (1.9%)	0.553	—
Postpartum hemorrhage	0 (0%)	1 (0.9%)	1.000	—

*Note:* The bold number/values indicate statistically significant calculations (*p* < 0.05).

### 3.3. Neonatal Outcomes

Overall, neonatal outcomes were comparable between groups, including 5‐min Apgar score, SGA, IUFD, macrosomia, congenital anomalies, and NICU admission. However, failed labor induction was associated with a higher incidence of low 1‐min Apgar scores compared with successful induction (25.5% vs. 10.4%; OR = 2.80; 95% CI, 1.16–6.79; *p* = 0.034). No cases of IUFD occurred in either group (Table 4).

**Table 4:** Neonatal outcomes in pregnancies with obesity that failed and successfully induced labor.

Neonatal outcome	Status of induction success		*p* value	OR value
	Failed *N*(%) 53 (100%)	Success *N*(%) 106 (100%)		
Apgar minute‐1 < 7	13 (25.5%)	11 (10.4%)	**0.034**	**OR 2.8** **CI 95% (1.16–6.79)** **contingency coefficient 18.3%**
Apgar Minute‐5 < 7	3 (5.7%)	5 (4.7%)	1.000	—
SGA	2 (3.8%)	6 (5.7%)	0.720	—
Macrosomia	4 (7.5%)	8 (7.5%)	1.000	—
Congenital anomalies	3 (5.7%)	4 (3.8%)	0.687	—
NICU admission	8 (15.1%)	7 (6.6%)	0.150	—

*Note:* The Bold number/values indicate statistically significant calculations (*p* < 0.05).

## 4. Discussion

This study identified primiparity, an unfavorable Bishop score, and greater gestational weight gain as significant factors associated with failed labor induction in obese pregnancies. Among these variables, primiparity emerged as the strongest predictor of induction failure. Consistent with our findings, Liu et al. reported a significantly higher cesarean delivery rate among obese primiparous women undergoing labor induction compared with their nonobese counterparts (23.49% vs. 12.29%; *p* = 0.002). This association has been hypothesized to reflect altered hormonal regulation, delayed cervical maturation, and increased adipose tissue accumulation in obese primiparous women, all of which may adversely affect the induction process [10]. This is consistent with a previous study conducted by Bjorklund et al. and Abdalla et al., which asserts that parity is the most significant risk factor for cesarean delivery following induction failure, irrespective of other variables [11, 12]. Primiparous women may face additional challenges related to limited labor experience, which can further compound the physiological and mechanical effects of obesity. This combination may substantially increase the likelihood of cesarean delivery [12, 13].

The Bishop score has been utilized for some time as a significant predictor of the success or failure of labor induction [14]. This study chose a PS criterion of < 3 versus ≥ 3 based on both current evidence and data analysis. According to Zelig et al. [15], obese women have poorer induction success rates and are more likely to have unfavorable cervical conditions, such as a low Bishop score (< 3) at the time of labor induction. Unfavorable cervical status significantly affects induction success, with obese women experiencing a 25% failure rate compared with nonobese women (14%, *p* = 0.001). For the initial study, pelvis scores were categorized into PS 2, PS 3, PS 4, and PS ≥ 5. Stepwise analysis showed a statistically significant correlation between PS and induction success (*p* = 0.001), with a modest level of association (26%), consistent with prior investigations. Subsequent evaluation of the study population showed that the largest proportion of successful inductions occurred among women with a PS of ≥ 3 (*n* = 58). To improve clinical application, pelvic ratings were divided into PS < 3 and PS ≥ 3. This category was statistically significant (*p* = 0.001) and gave a more pragmatic clinical decision‐making criterion. This study supports a PS of ≥ 3 as a clinically relevant reference point for labor induction, based on prior evidence and the distribution of successful inductions in the cohort. Obese women were more likely to have a low Bishop score, which was correlated with increased risk of labor induction failure [14]. Obesity is associated with hormonal alterations and changes in uterine tone that may impair cervical ripening. Experimental studies have demonstrated reduced frequency and intensity of uterine contractions in obese women, potentially mediated by inhibitory effects of elevated maternal leptin and cholesterol levels, as well as altered calcium signaling, leading to diminished myometrial activity [12, 16].

We also discovered that maternal weight gain, especially excessive weight gain, was one of the factors that was associated with the failure of labor induction in our study. Excessive maternal weight gain has been defined according to the criteria set forth by the IOM in 2009. Recommended weight gain is determined by the mother′s BMI at the onset of pregnancy. Pregnant women with a normal BMI are advised to achieve a total weight gain of 11–15 kg throughout gestation. A systematic review and meta‐analysis demonstrated that excessive gestational weight gain may contribute to labor induction failure, particularly when weight gain is sufficient to advance maternal obesity to a higher BMI category [8]. Prior studies have reported a stronger association between BMI and cervical ripening failure in obese women at delivery compared with women of normal weight. This finding has been attributed to an increased volume of distribution at delivery relative to pre‐pregnancy BMI, underscoring the relevance of assessing BMI at the time of labor induction. Our findings are consistent with those of previous investigations [8]. Previous studies have shown that the likelihood of labor induction failure increases with higher BMI and advancing obesity class. However, this association could not be confirmed in the present study, as only a single case of Class III obesity was observed in the cohort, limiting the ability to evaluate its independent effect, despite evidence identifying Class III obesity as a risk factor for induction failure [17, 18].

Our research revealed that the group that failed labor induction had a higher incidence of severe preeclampsia, a longer duration of hospitalization, and a higher incidence of cesarean section in comparison to the successful group in terms of maternal outcomes. This is consistent with the findings from other studies [19, 20]. Obese women have a more than threefold increased risk of preeclampsia when their BMI exceeds 30 kg/m2 [21, 22]. In this study, preeclampsia and severe preeclampsia were predominantly present before the initiation of labor induction, although a proportion of cases developed during the induction process. Previous evidence indicates that a preconception weight reduction of approximately 4.5 kg may decrease the risk of preeclampsia by up to 50%. Furthermore, a systematic review has reported that obese women have a 3‐ to 10‐fold increased risk of preeclampsia, supporting a direct association between maternal obesity and hypertensive disorders of pregnancy [21, 22]. Maternal obesity is associated with endothelial dysfunction, a key pathophysiologic mechanism underlying preeclampsia. Increased insulin resistance, which commonly accompanies obesity, is also considered a major contributor to the development of preeclampsia [22]. Women with severe preeclampsia had a higher induction failure rate than women without preeclampsia, according to research conducted by Kyo et al. The use of magnesium sulfate and an increase in maternal weight were the determining factors [23].

In this study, all women who experienced failed labor induction—defined as failure to progress to the active phase after one induction series—subsequently underwent cesarean delivery. In contrast, cesarean delivery occurred in 5.7% of women in the successful induction group, primarily due to fetal distress or labor arrest precluding continued vaginal delivery. Consistent with these findings, prior studies have reported an increased risk of cesarean delivery among obese women, largely attributable to higher rates of induction failure [24]. The markedly higher rate of cesarean delivery in the failed induction group largely explains the longer duration of hospitalization compared with women who achieved successful induction. This difference was further influenced by the higher incidence of preeclampsia and severe preeclampsia in the failed induction group, conditions that require prolonged postpartum monitoring. Consistent with prior reports, maternal obesity and its associated complications have been linked to longer hospital stays and increased healthcare utilization, with admissions exceeding 5 days occurring more frequently among women with BMI > 30 kg/m [1]. Notably, the incidence of postpartum hemorrhage did not differ significantly between groups [25].

This study also demonstrated that failed labor induction was associated with a higher incidence of low 1‐min Apgar scores compared with successful induction. The 1‐min Apgar score reflects the newborn′s immediate adaptation to extrauterine life and serves as an indicator of intrapartum condition and the neonatal response to the stress of birth, guiding the need for initial resuscitative interventions. Consistent with prior findings, Jeganathan et al. reported that maternal obesity and diabetes were independently associated with a greater likelihood of Apgar score recovery compared with normal maternal weight and absence of diabetes [26]. Consistent with these findings, no significant difference was observed in 5‐min Apgar scores between obese pregnancies with failed versus successful labor induction. In this cohort, low 1‐min Apgar scores among women with failed induction may also reflect the presence of underlying pregnancy‐related complications rather than persistent neonatal compromise [26].

Maternal obesity has been associated with an increased risk of congenital anomalies. Stothard et al. reported a higher likelihood of birth defects, including neural tube defects, among pregnancies complicated by maternal obesity. In the present study, congenital anomalies were identified in seven cases (4.4%), including multiple congenital anomalies (*n* = 2), anorectal malformations (*n* = 2), spina bifida (*n* = 1), diaphragmatic hernia (*n* = 1), and congenital talipes equinovarus (*n* = 1) [27]. However, the occurrence of congenital anomalies did not differ significantly according to labor induction success status.

### 4.1. Study Limitations

This study possesses multiple limitations that warrant acknowledgment. The cross‐sectional approach restricts causal inference between identified risk factors and induction failure. Secondly, data were obtained from medical records, which may be susceptible to information bias and residual confounding from unmeasured variables, including cervical ripening techniques, provider decision‐making, and patient preferences. The restricted sample size, especially the few number of patients with Class III obesity, diminishes the capacity to evaluate the independent impact of extreme obesity on induction results. The implementation of a singular standardized induction methodology may not account for practice variances in other contexts; hence, potentially constraining external validity.

## 5. Conclusions

In obese pregnancies at term, primiparity, a low Bishop score, and high gestational weight gain are independently correlated with unsuccessful labor induction. Induction failure is additionally associated with elevated incidences of severe preeclampsia, cesarean birth, prolonged hospitalization, and inadequate early neonatal adaptation. These results highlight the necessity of meticulous patient selection, enhancement of cervical preparation, and proactive counseling when contemplating labor induction in obese women to enhance maternal and newborn outcomes.

## Funding

No funding was received for this manuscript.

## Conflicts of Interest

The authors declare no conflicts of interest.

## Data Availability

The data that support the findings of this study are available from the corresponding author upon reasonable request.
